# Recent genetic, phenetic and ecological divergence across the Mesoamerican highlands: a study case with *Diglossa baritula* (Aves: Thraupidae)

**DOI:** 10.7717/peerj.16797

**Published:** 2024-03-22

**Authors:** Alondra K. Terrones-Ramírez, Sahid M. Robles-Bello, Melisa Vázquez-López, Sandra M. Ramírez-Barrera, Luz E. Zamudio-Beltrán, Anuar López López, Maria del Coro Arizmendi, Ana Paula Durán-Suárez del Real, Luis E. Eguiarte, Blanca E. Hernández-Baños

**Affiliations:** 1 Departamento de Biología Evolutiva, Facultad de Ciencias, Universidad Nacional Autónoma de México, Ciudad de México, CdMx, Mexico; 2 Posgrado en Ciencias Biológicas, Universidad Nacional Autónoma de México, Ciudad de México, CDMX, México; 3 Laboratorio de Ecología, UBIPRO Facultad de Estudios Superiores Iztacala, Universidad Nacional Autónoma de México, Tlalnepantla, Estado de México, Mexico; 4 Departamento de Ecología Evolutiva, Instituto de Ecología, Universidad Nacional Autónoma de México, Ciudad de México, CdMx, Mexico

**Keywords:** Coloration, Ecological niche, Flowerpiercer, Isthmus of Tehuantepec, Mexico, Morphology, NextRAD, Pleistocene climatic events, Speciation

## Abstract

The topographical, geological, climatic and biodiversity complexity of Mesoamerica has made it a primary research focus. The Mesoamerican highlands is a region with particularly high species richness and within-species variation. The Cinnamon-bellied Flowerpiercer, *Diglossa baritula* (Wagler, 1832), is a species endemic to the Mesoamerican highlands, with three allopatric subspecies currently recognized. To characterize divergence within this species, we integrated genomics, morphology, coloration and ecological niche modeling approaches, obtained from sampling individuals across the entire geographic distribution of the species. Our results revealed a clear genomic divergence between the populations to the east versus the west of the Isthmus of Tehuantepec. In contrast to the genomic results, morphology and coloration analyses showed intermediate levels of differentiation, indicating that population groups within *D. baritula* have probably been under similar selective pressures. Our morphology results indicated that the only sexually dimorphic morphological variable is the wing chord, with males having a longer wing chord than females. Finally, ecological data indicated that there are differences in ecological niche within *D. baritula*. Our data suggest that *D. baritula* could contain two or more incipient species at the intermediate phase of the speciation continuum. These results highlight the importance of the geographical barrier of the Isthmus of Tehuantepec and Pleistocene climatic events in driving isolation and population divergence in *D. baritula*. The present investigation illustrates the speciation potential of the *D. baritula* complex and the capacity of Mesoamerican highlands to create cryptic biodiversity and endemism.

## Introduction

The profound complexity and biodiversity of Mesoamerica have positioned it as a focal point of primary research. The Mesoamerican highlands, characterized by a fragmented distribution influenced by environmental factors such as altitude, temperature, and humidity (Hernández-Baños et al., 1995; Barrantes, Iglesias & Fuchs, 2011), exhibit particularly high species richness and intraspecific variation (Myers et al., 2000; Pérez-Emán, 2005; Sánchez-Ramos et al., 2018). The presence of isolated patches housing high-altitude taxa facilitates substantial phenetic and genetic divergence among populations (García-Moreno et al., 2004).

The genus *Diglossa* represents one of many evolutionary radiations that have accumulated species over the past eight million years (Fig. S1, Mauck & Burns, 2009; Barker et al., 2015). This genus has a neotropical distribution, with most species inhabiting South America. The early-diverging branches are distributed in the Northern Andes, suggesting that the *Diglossa* lineage is of the Andean biogeographic origin. There were two major dispersal events, one from the Northern Andes to the tepuis of Venezuela during the Miocene, and the other from the Northern Andes to Central America in the Pliocene (Hackett, 1995; Mauck & Burns, 2009). This second dispersal event can explain the presence of the only two Mesoamerican species—*D. baritula* and its sister species, *D. plumbea*. Thus, *Diglossa* species and their populations have a wide dispersal capacity as well as potential for speciation.

The Cinnamon-bellied Flowerpiercer, *Diglossa baritula* (Wagler, 1832), is distributed in the Mesoamerican highlands (Lauck, 2020, Fig. 1A) and belongs to a genus that is considered one of the Neotropical evolutionary radiations (Vuilleumier, 1969). Both the genus *Diglossa* and the *D. baritula* complex have had an array of taxonomic arrangements (Hellmayr, 1935; Friedmann, Griscom & Moore, 1950; Skutch, 1954; Monroe Jr, 1968; Vuilleumier, 1969; Bock, 1985; Isler & Isler, 1987; Sibley & Monroe, 1990; Dickinson, 2003; Remsen et al., 2008; Tables S1 and S2). The genus *Diglossa* (Aves: Thraupidae) presents a wide variation in (i) plumage coloration patterns (Mauck & Burns, 2009; Lauck, 2020), (ii) the presence and absence of sexual dichromatism, (iii) body size (Lauck, 2020), (iv) bill size and shape (Mauck & Burns, 2009), (v) diet composition (Lauck, 2020; Schondube & Martínez del Rio, 2003) and (vi) altitudinal range (from coast to highlands) (Gutiérrez-Zuluaga et al., 2021). Furthermore, there are species complexes whose phylogenetic relationships remain unresolved (Gutiérrez-Zuluaga et al., 2021). This biological heterogeneity has evolved over a short time scale and small geographic range (Barker et al., 2015; Fig. S1).

**Figure 1:**
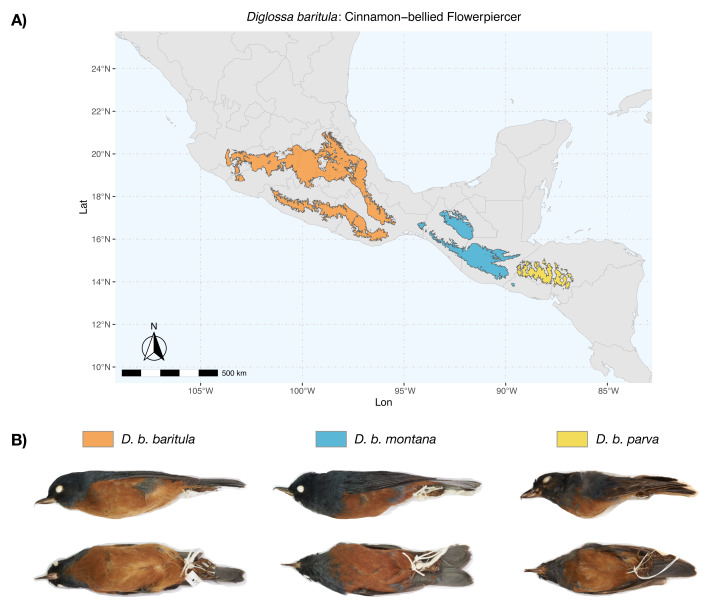
Geographic distribution of *D. baritula*. (A) Geographic distribution of *D. baritula* showing the allopatric subspecies represented by different colors: *D. b. baritula* in orange, *D. b. montana* in blue and *D. b. parva* in yellow. (B) Male specimens of each subspecies in lateral and ventral position, respectively. Photographs provided by Sahid M. Robles-Bello. The shape used for representing the current geographic distribution for the species was taken from the CONABIO geoportal: http://www.conabio.gob.mx/informacion/gis/ (Navarro-Sigüenza et al., 2018).

*Diglossa baritula* (Cinnamon-bellied Flowerpiercer) has three allopatric subspecies that are currently recognized based on disjunct distributions and qualitative descriptions of male plumage (Howell & Webb, 1995; Lauck, 2020). *Diglossa baritula baritula* (Wagler, 1832) occurs in Mexico, from southern Jalisco to west of the Isthmus of Tehuantepec; it has cinnamon-rufous ventral coloration that extends from the throat to the tail coverts. *Diglossa baritula montana* (Dearborn, 1907) is distributed from east of the Isthmus of Tehuantepec to southern El Salvador; the throat is gray and ventral underparts are deeper cinnamon-rufous. *Diglossa baritula parva* (Griscom, 1932) is restricted to Honduras and northwestern Nicaragua; it is similar to *D. b. montana*, but smaller, with a shorter and slenderer bill (Fig. 1). In addition, most studies on *D. baritula* have been focused on their ecology and behavior, mainly related to the unusual feeding behavior of this species, which robs nectar from flowers that are generally pollinated by hummingbirds (Arizmendi, Domínguez & Dirzo, 1996; Schondube & Martínez del Rio, 2003).

Here, we present a multidisciplinary study of the populations of *D. baritula* and the results obtained allow us to have a first perspective about the natural history of this species. The aim of this research is to understand the phylogenetic relationships at population level for the species; describe the pattern of morphology, coloration and environmental variation; and relate these patterns with the phylogenetic hypothesis. We expected to find high levels of genetic, phenetic and environmental variation, since the *D. baritula* complex is a resident species with allopatric distribution and belongs to an adaptive radiation.

## Materials and Methods

### Genomic data

We extracted total genomic DNA from six tissue samples comprising the three currently recognized subspecies (three from *D. b. baritula*, two from *D. b. montana* and one from *D. b. parva*; Table S3, Fig. S2A) using the Epicentre MasterPure kit (Epibio). Tissue samples were taken from the “Museo de Zoología, Facultad de Ciencias UNAM” (collection permit: Instituto Nacional de Ecología, SEMARNAT, Mexico FAUT-0169), as well as samples donated by other institutions. We verified the DNA quality with gel electrophoresis and quantified the DNA concentration using a Qubit 3 fluorometer. We included one sample from *Diglossa plumbea* as the sister group and one sample from *Euphonia hirundinacea* as the outgroup. Genomic DNA was submitted to SNPsaurus (http://snpsaurus.com/) to construct NextRAD genotyping-by-sequencing libraries as in Russello et al. (2015). First, genomic DNA was fragmented with Nextera reagent (Illumina, Inc., San Diego, CA, USA), and short adapter sequences were ligated to the ends of the fragments. Fragmented DNA was then amplified for 27 cycles at 74 ° C, with one of the primers matching the adapter and extending 10 nucleotides into the genomic DNA with the selective sequence GTGTAGAGCC. Sequencing was performed on a HiSeq 4000 with a lane of 150 bp reads (University of Oregon). Raw sequence reads were deposited in the Sequence Read Archive (SRA) at the National Center for Biotechnology Information (NCBI) under BioProject PRJNA901463 with accession numbers SRR22282685 –SRR22282692.

The quality of the raw reads was verified with FastQC v. 0.11.9 (Andrews, 2019). We assembled demultiplexed reads into sequence alignment through ipyrad v 0.9.53 (Eaton & Overcast, 2020) using the default settings with the following exceptions: reads were mapped to a *D. b. baritula* draft genome assembly (Licona-Vera, 2022, unpublished data; Table S3; SRA accession ID: SRR23341254, BioProject accession ID: PRJNA931586), CACATCTCGG for restriction overhang, the cluster threshold was set to 0.93 (McCartney-Melstad, Gidis & Shaffer, 2019) and the parameters used to filter out poor quality reads were changed according to FastQC results. To assess the phylogenetic relationships among individuals, we used a Maximum Likelihood (ML) approach, under the GTR+Gamma evolutionary model, and nodal supports were accessed using 1,000 ultrafast bootstrap replicates using IQTree v. 1.6.12 (Nguyen et al., 2015) on the CIPRES Science Gateway v.3.3 (Miller, Pfeiffer & Schwartz, 2010). FigTree v. 1.4.4 (Rambaut, 2018) was used to display the tree and the bootstrap values of each node.

SNPs were further filtered from our initial dataset using the R package SNPfiltR (DeRaad, 2022) in order to select only biallelic and unlinked loci. This resulted in a reduced dataset of 33,702 loci, which was then used for further analysis. To assess potential gene flow across subspecies, we used the program Dsuiteb v0.2 r17 (Malinsky, Matschiner & Svardal, 2021) to calculate Patterson’s *D* statistic, which is the test statistic for the ABBA–BABA test (Green et al., 2010; Durand et al., 2011), following the authors’ scripts (https://github.com/millanek/Dsuite). The ABBA-BABA test can be performed on any four-taxon phylogeny in the form (((P1,P2),P3),O) with P1 to P3 being ingroups and O being the outgroup. The test was conducted with *D. plumbea* as the outgroup. Positive values of the *D* statistic imply that there is gene flow between P2 and P3, and negative values imply gene flow between P1 and P3. The test counts the number of ABBA patterns (where P2 and P3 share a derived allele) and BABA patterns (where P1 and P3 share a derived allele).

To construct species trees with our biallelic, unlinked SNP dataset we used the coalescent-based SNAPP algorithm implemented in the software BEAST v.2 (Bouckaert et al., 2014). Mutation rates were estimated empirically, and other parameter priors were left at the defaults. MCMC was run for 10,000,000 iterations, discarding the first 10% as burn-in. Trees and parameter estimates were sampled every 1,000 iterations, and the resulting trees were visualized using DensiTree (Bouckaert, 2010).

Divergence times were estimated using BEAST v1.10.4 (Bouckaert et al., 2014); we used a Bayesian relaxed clock with an uncorrelated lognormal distribution (Drummond et al., 2006; Li & Drummond, 2012) and a Yule model as a speciation prior. We assigned two calibration nodes based on secondary calibrations: the split between Thraupidae and Cardinalidae (12.7772 Mya with a 95% HPD of 11.4984–14.4482; (Barker et al., 2015)) and the split between *D. baritula* and its sister species, *D. plumbea* (0.4401 Mya with a 95% HPD of 0.2848–0.6613; Barker et al., 2015). We performed two independent runs with 10 million generations each, with parameters sampled every 1,000 generations, and discarding the first 25% of generations as burn-in. Convergence from the independent runs, ESS values above 200 and burn-in value were confirmed in Tracer v1.6 (Rambaut et al., 2014). Replicate results were combined in LogCombiner v1.8.2 (Drummond et al., 2012). The resulting posterior sample of trees was summarized in a Maximum Clade Credibility (MCC) tree using TreeAnnotator v1.8.2 (Drummond et al., 2012), obtaining mean divergence times with 95% highest posterior density intervals. The consensus species tree with the divergence times was visualized in FigTree v1.4.4 (Rambaut, 2018).

### Coloration and morphological data

To quantify color variation, we measured the reflectance spectra of six plumage patches (upper back, lower back, throat, breast, upper belly and lower belly) of 85 males (Table S3, Fig. S2B) over the avian visual range (300–700 nm). Three measurements were taken per patch. We used an Ocean Optics USB2000 spectrophotometer with a PX-2 pulsed xenon light source and a bifurcated fiber optic probe (Ocean Optics, Dunedin, FL, USA). The measuring probe was directed perpendicularly to the feather surface and ambient light was blocked with a drilled rubber stopper surrounding the tip of the probe. The spectrophotometer was calibrated with a white standard (WS-2, Ocean Optics, Dunedin, FL, USA). Prior to analysis, the three measurements were averaged and smoothed (smoothing parameter of 0.25) to remove electrical noise. To visually assess variation, we constructed reflectance curves comparing each plumage patch of the subspecies. Lastly, to determine significant differences we calculated chromatic just noticeable distances (JND; Vorobyev & Osorio, 1998) for each patch assuming a Weber fraction of 0.1 for the long-wavelength sensitive cone. The visual system we used was the spectral sensitivity data from the Blue Tit *Cyanistes caeruleus* (based on Hart et al., 2000), since it is the phylogenetically closest species available in the package. A value of 1 JND is considered the threshold that represents the distance in the perceptual color space at which two colors would be visually discernible, thus if the distances (including their 95% confidence intervals) have a value >1 JND, color differences are discernible. All spectral processing was performed using package pavo 2.1.0 (Maia et al., 2019) in R v. 4.0.2 (R Core Team, 2020). The ventral coloration of females is patchy (without homogeneous coloration), which makes it difficult to reach adequate measurement repeatability; we therefore only analyzed male coloration.

To examine the morphological variation in *D. baritula*, we obtained five standard body measurements from 163 adult specimens (93 males and 70 females) from 75 localities housed in nine skin collections (Table S3, Fig. S2C). Measurements were taken with a digital caliper to the nearest 0.01 mm by the first author. The measurements were: bill length (BL, from anterior edge of skull to the tip of the culmen), bill width (BW, culmen width at the nostril), tarsus length (TL) and wing cord (WC, from the carpal joint to the tip of the longest primary), following Baldwin, Oberholser & Worley (1931), and bill hook length (BHL; HL in Mauck & Burns (2009)). Wing and tarsus measurements were taken from the right side of each specimen. We included only adult individuals of known sex. All individuals were measured three times and those values were averaged. We first evaluated the normality and the homogeneity of variances with Shapiro–Wilk test and *F* tests, respectively. Then, we determined if measurements differed between males and females with *t*-tests. Since we only found significant differences between sexes in WC, we analyzed males and females separately for that variable but considered the sexes together for the rest of the morphological traits. Differences among subspecies in the morphological variables were examined using one-way ANOVAs followed by Tukey’s *post hoc* comparisons. Descriptive statistics included means and standard deviations. We performed a principal components analysis (PCA) and obtained PC scores for each individual. We also plotted individuals’ scores on PC1 *versus* PC2 to visualize placement of the subspecies in morphospace. All tests were performed in R v. 4.0.2 (R Core Team, 2020) and the significance level was set at 0.05.

### Ecological niche modeling and niche overlap

We used ecological niche models (ENM) to simulate the potential distribution of past refugia based on current occurrence records for the species and to determine the degree of overlap in the environmental space across subspecies. We obtained records of occurrence from the collection locations of the specimens used for our genomic, coloration and morphology sampling, as well as from the Global Biodiversity Information Facility (http://www.gbif.org/) (Table S3, Fig. S2D). To avoid spatial autocorrelation, we used 18.53 Km as the threshold distance for duplicated data with the R package Nichetoolbox (Osorio-Olvera et al., 2016). This resulted in a total of 173 records for *D. b. baritula*, 72 for *D. b. montana* and 43 for *D. b. parva*.

We used current and past bioclimatic layers from the WorldClim database (Hijmans et al., 2005). Past scenarios were projected into three different periods: the Last InterGlacial (140–120 kya), the Last Glacial Maximum (21 kya), and the Mid Holocene (6 kya). We selected the most important variables from the first three principal components from PCA (Fig. S3) that were not highly correlated based on a Pearson correlation Test (*r* < 0.75, see Fig. S3). We used the ellipsenm R package (Cobos et al., 2020) to define the accessible area for the species (“M” area). Past projections were performed in Maxent v3.4.1 with 10 cross validation replicates. We selected a conservative threshold corresponding to the maximum training sensitivity plus the specificity logistic threshold.

To compare the current environmental spaces occupied by each subspecies, we used the ecospat R package (Di Cola et al., 2017)). To reduce the environmental space to informative variables only, we transformed it into a two-dimensional space defined by the first two principal components (Fig. S4). We performed niche equivalency and similarity tests, and used as the study area the accessible area for the species (“M” area). Two metrics were obtained using 100 iterations for simulated data: Schoener’s D and Hellinger’s I (Broennimann et al., 2012; Warren, Glor & Turelli, 2008), which range from 0 (no overlap) to 1 (complete overlap). Niche overlap analyses generated with occurrence data were compared with pseudo-replicate models generated with randomly redistributed occurrence data. We tested the niche overlap analyses using the “lower” option. In this procedure, the smaller the empirically observed D and I values compared to those generated by the pseudo-replicates, the more significant the niche difference, thus the null hypothesis of niche similarity is rejected. The raw data and scripts generated for coloration, morphology and ecological niche modeling analysis are available in DOI: 10.5281/zenodo.8019104.

## Results

### Genomics

After quality filtering, our data set contained 1,278,209 to 5,344,377 reads for eight samples with an average of 3,897,579 reads per individual. Using the reference assembly approach on ipyrad produced 43,704 consensus loci, 178,160 SNPs and 5,827,151 pb. The missing sites were 18.15% for the SNPs matrix and 22.73% for the sequence matrix.

**Figure 2:**
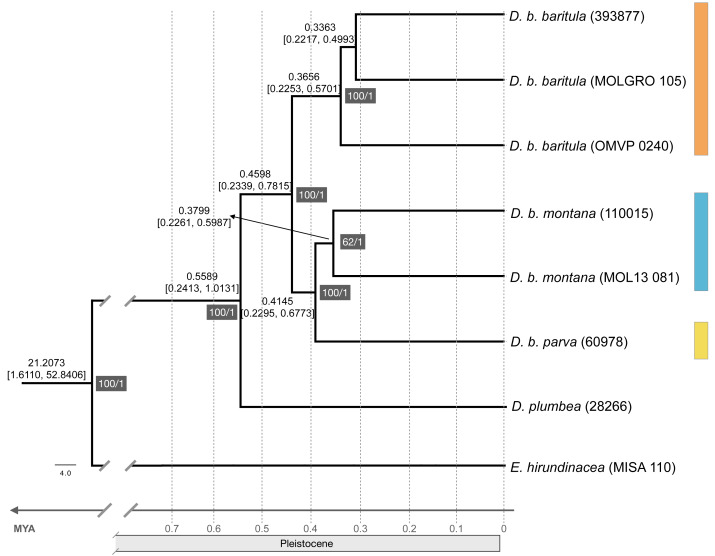
Maximum likelihood phylogenomic tree and divergence times. Maximum likelihood phylogenomic tree obtained from 44,739 RADseq loci representing phylogenetic relationships among clades in the *Diglossa baritula* complex: *D. b. baritula* in orange, *D. b. montana* in blue and *D. b. parva* in yellow. The numbers highlighted in gray represent support values, on the left are the bootstrap values obtained from the maximum likelihood tree and on the right are the posterior probability values obtained for the molecular clock. Numbers above nodes represent age of the node. *Diglossa plumbea* and *Euphonia hirundinacea* are used as sister group and outgroup, respectively.

The ML tree recovered the *D. baritula* complex as a well-supported monophyletic lineage (100% bootstrap support). The deepest split was between two strongly supported clades: the West-IT Group (100%) included all individuals of *D. b. baritula* which are distributed west of the Isthmus of Tehuantepec, and the East-IT Group (100%), which included the *D. b. montana* and *D. b. parva* individuals, which are both distributed east of the Isthmus of Tehuantepec (See Fig. 2). Individuals from the subspecies with “cinnamon throat males” clustered together, and samples from the subspecies with “gray-throated males” group together as well. In general, the topology revealed phylogeographic structure within the *D. baritula* complex. Conversely, the SNAPP species tree analysis based on 33,702 unique RAD loci did not support *D. baritula* complex as a monophyletic clade with two clades as obtained in the ML tree. The sister species, *D. plumbea*, is embedded within the complex, and one individual from *D. b. baritula* is closely related with the outgroup *E. hirundinacea* (Fig. S5). The time-calibrated tree estimated that *D. baritula* diverged from its sister species, *D. plumbea*, ∼0.5589 Mya. Divergence between the two main clades, West-IT Group and East-IT Group, occurred ∼0.4598 Mya during the Pleistocene (Fig. 2). Lastly, the ABBA-BABA test did not detect a significant signal of gene flow (*P* > 0.05, see Table 1).

**Table 1:** Results of ABBA-BABA tests in *D. baritula*. The outgroup was *D. plumbea.*

**P1**	**P2**	**P3**	***D*** ** statistic**	***Z*-score**	***p*-value**	**f4-ratio**	**ABBA**	**BABA**
*parva*	*montana*	*baritula*	0.00201383	0.0781593	0.937701	0.00227271	430.188	428.458

### Coloration and morphological analyses

**Figure 3:**
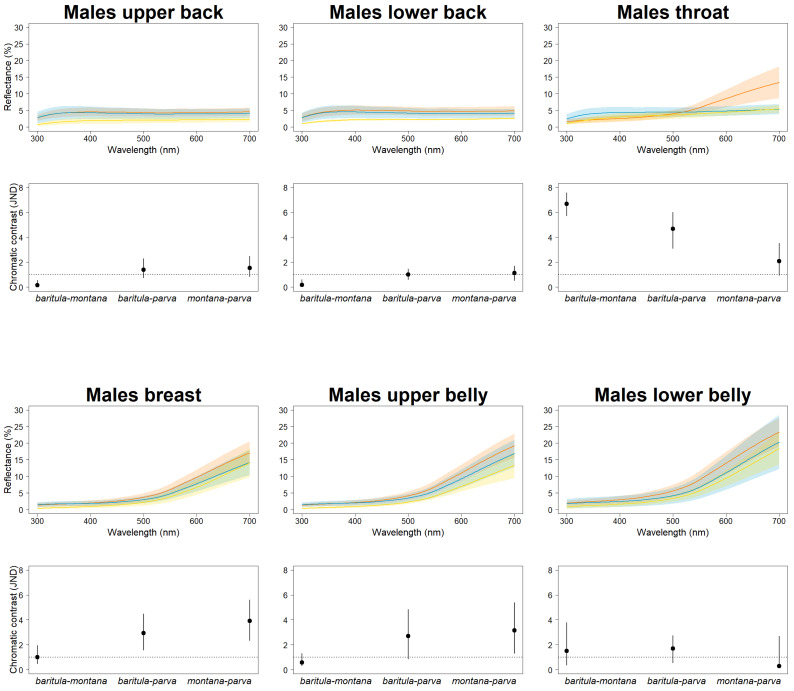
Color differences of *D. baritula* males. The top panel shows the mean spectral reflectance curves for six plumage patches of adult males. The shaded area around each line indicates standard error of the mean: in orange *D. b. baritula*, in blue *D. b. montana* and in yellow *D. b. parva*. The bottom panel shows the color distances in units of chromatic contrast (just noticeable differences, JND). Points and bars represent the mean values and 95% confidence intervals, respectively. The horizontal dashed line indicates the discriminability threshold (JND of 1); color comparisons with JND greater than 1 are considered perceptually distinguishable among subspecies.

In our JNDs analyses, we found magnitudes of difference greater than the 1 JDN threshold (including the 95% confidence intervals) in plumage color among the three named subspecies in both sexes for most feather patches we measured, *i.e.,* we detected statistically and perceptually significant differences among subspecies. Differences in color on the slate-colored back feathers are borderline nonsignificant, with the bulk of chromatic variation being observed on the reddish ventral patches (Fig. 3). This is particularly noticeable in the *baritula* subspecies, where the reddish ventral coloration extends up to the throat and shows a distinct slope in the corresponding spectral shape. The throat patch was significantly different between the eastern group and the western group.

**Figure 4:**
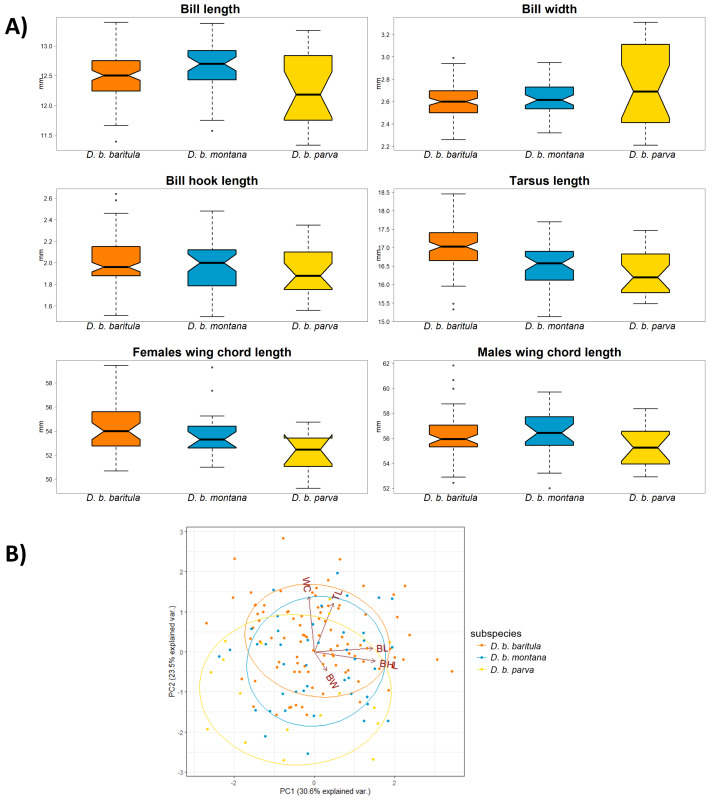
Morphological variation in *D. baritula* based on five measured morphological variables. (A) Boxplots of morphological variables. Wing chord was analyzed separately for each sex, since our results indicated differences between sexes (see results and Table 2). Boxes span the first and third quartile of data with the median as a horizontal line, and whiskers (vertical dashed lines) represent the data rango, excluding outliers. (B) Principal component biplot of morphological variables for the first two axes. Ellipses are graphical tools that represent the 95% confidence intervals of the principal component scores. Arrowed lines show direction and magnitude of each variable. For summary statistics, see Table 2. In orange *D. b. baritula*, in blue *D. b. montana* and in yellow *D. b. parva*. BL, bill length; BW, bill width; BHL, beak hook length; TL, tarsus length and WC, wing cord.

We found no significant morphological sexual dimorphism in this complex in four of the five morphometric variables measured. Only wing cord (WC) showed statistically significant differences between sexes (Table 2). For the measurements of bill length (BL), bill width (BW), bill hook length (BHL) and tarsus length (TL) we found significant differences among subspecies. However, the only measurement that showed a significant difference between the East-IT and West-IT Groups was TL (Fig. 4A, Table 2).

**Table 2:** Mean and ANOVA results. Mean and ANOVA to test differences between subspecies and PCA values.

**Measurement**	**Sex**	**Mean ± SD**			***F***	***df***	***p*** **-value**	**PC1**	**PC2**
		*D. b. baritula*	*D. b. montana*	*D. b. parva*				30.60%	23.50%
Bill length	Both	12.48 ± 0.40	12.65 ± 0.41	12.33 ± 0.60	4.163	2	**<0.05**	0.67	0.04
Bill width	Both	2.61 ± 0.15	2.63 ± 0.15	2.74 ± 0.37	4.467	2	**<0.05**	0.14	−0.24
Bill hook length	Both	2 ± 0.20	1.97 ± 0.22	1.92 ± 0.22	1.523	2	0.221	0.69	−0.12
Tarsus length	Both	17.05 ± 0.57	16.55 ± 0.58	16.35 ± 0.61	20.42	2	**<0.001**	0.21	0.63
Wing chord	Females	54.09 ± 2.1	53.72 ± 2.04	52.22 ± 1.81	3.063	2	0.053	−0.06	0.72
Wing chord	Males	56.28 ± 1.77	56.46 ± 2	55.51 ± 1.72	1.381	2	0.257		

**Notes.**

Significant differences are indicated in bold.

*df*, degrees of freedom.

The PCA analyses indicated that the first two principal components explained 54.1% of the observed variation. Bill hook length and bill length had the highest correlation with principal component 1 (PC1, representing 30.6% of variance), and tarsus length was correlated with principal component 2 (PC2, 23.5%; Table 2). The PCA scores showed that the three subspecies had a high degree of overlap. The analyses did not show differences between the East-IT Group and the West-IT Group (Fig. 4B).

### Ecological niche modeling and Niche overlap

**Figure 5:**
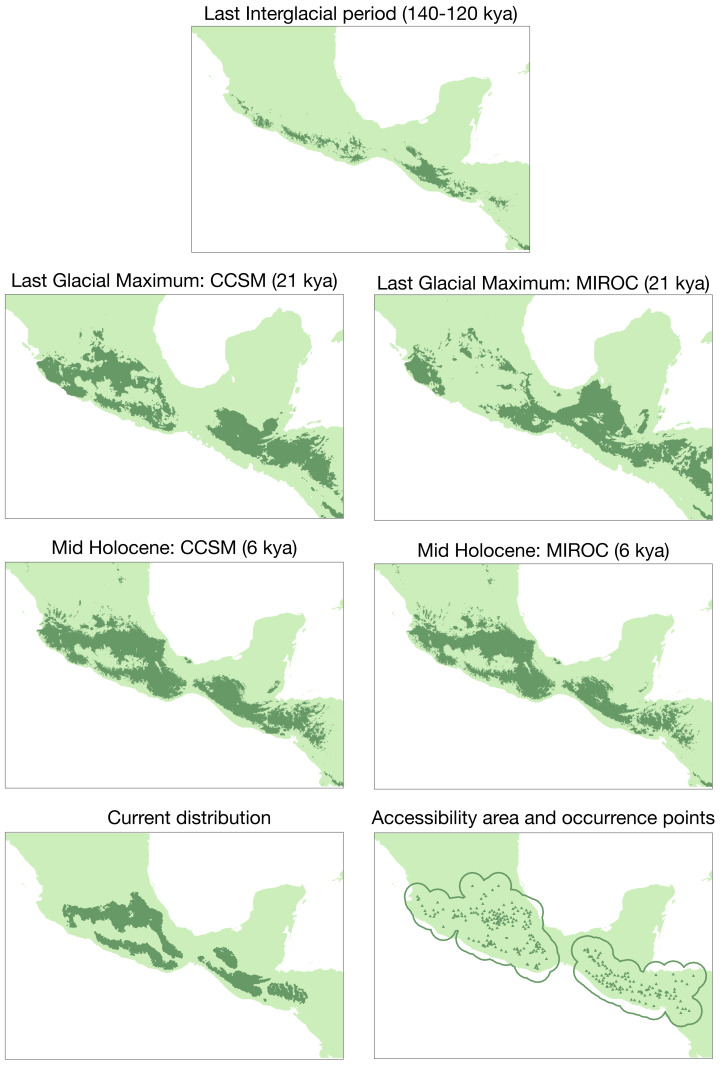
Ecological niche modeling for the *Diglossa baritula* complex under different past scenarios. Bottom left: current geographic distribution for the species ((Navarro-Sigüenza et al., 2018); CONABIO geoportal: http://www.conabio.gob.mx/informacion/gis/). Bottom right: distribution of occurrence points used for the modelling process, and accessible area (“M”).

In the past projection analysis, the first three principal components explained most of the variation for the species (PCI: 41.2%, PC2: 29%, PC3: 14.1%; Fig. S3). According to this PCA and the correlated variables, we selected the following bioclimatic variables: bio4 (temperature seasonality), bio5 (max. temperature of warmest month), bio6 (min. temperature of coldest month), bio7 (temperature annual range), bio12 (annual precipitation), bio14 (precipitation of driest month), and bio15 (precipitation seasonality). The average test AUC for the replicate runs was 0.922, and the standard deviation was 0.017. The conservative threshold value chosen was 0.1885. Results from niche models are in Fig. 5. There are differences across past scenarios where expansion periods had occurred from the Last InterGlacial period to the Last Glacial Maximum (LGM) and Mid Holocene (MH) periods. Also, there is evidence of contraction from the intermediate periods (LGM and MH) to the present time.

**Figure 6:**
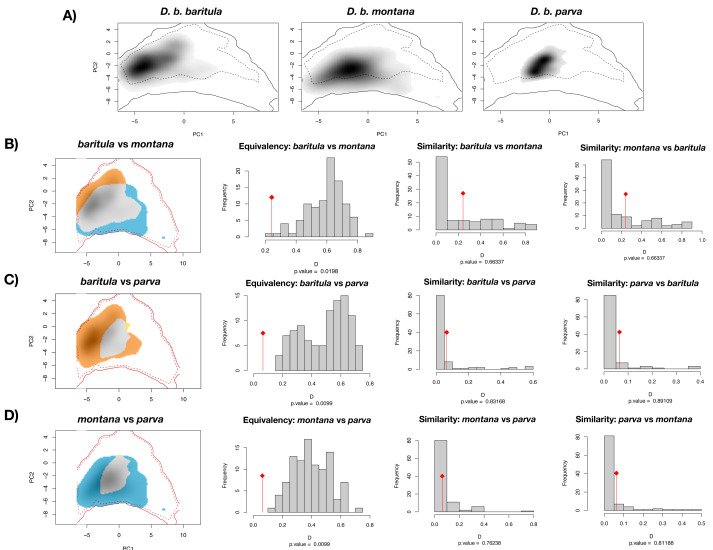
Ecological niche overlap results. Current ecological niche overlap analysis. (A) Environmental space for the three subspecies described for the *Diglossa baritula* complex: *D. b. baritula*, *D. b. montana* and *D. b. parva*. (B) Comparisons between *baritula* and *montana* subspecies. (C) Comparisons between *baritula* and *parva* subspecies. (D) Comparisons between *montana* and *parva* subspecies. Central histograms show results of Equivalency and Similarity tests. Gray columns represent null distributions of D values. Red diamonds and lines represent observed values of D.

The PCA obtained from the niche overlap analysis indicated a contribution of 48.7% for the first principal component and 23% for the second (Fig. S4). In Fig. 6, environmental space plots showed the dimensions of visual overlap for the comparisons across subspecies. The resulting D and I metrics were: (1) *baritula vs montana*: *D* = 0.24148, *I* = 0.35606; (2) *baritula vs parva*: *D* = 0.06433, *I* = 0.23520; and (3) *montana vs parva*: *D* = 0.06209, *I* = 0.23838. The observed D values are very low (in the cases of baritula *vs* parva and monatana *vs* parva they are practically zero, which could indicate that the niches do not overlap). In the identity tests, the null hypotheses of niche equivalency are rejected (*P* < 0.05), revealing that the niches are not identical. In the similarity tests, *P* values obtained in similarity tests (*P* > 0.05) indicated that the null hypothesis of niche divergence must be accepted and therefore, the niche overlap is less similar than random. However, in these niche similarity analyses the observed niche overlap scores fell into the simulated niche overlap scores, suggesting that their niche is not significantly different (Fig. 6). Overall, these results suggest that the subspecies occupy different niches.

## Discussion

*Diglossa baritula* (Cinnamon-bellied Flowerpiercer) is a species comprising three currently recognized allopatric subspecies. Here, we performed different analyses to obtain a phylogeny of the populations and describe the pattern of morphology, coloration and environmental variation. Our phylogenetic tree shows a well-supported deep divergence between populations on either side of the Isthmus of Tehuantepec. The West-IT Group is a monophyletic clade that comprises all individuals of *Diglossa baritula baritula*, while the East-IT Group is another monophyletic lineage that contains *Diglossa baritula montana* and *Diglossa baritula parva*. Individuals from the subspecies with “cinnamon-throated males” form one group, and samples from the subspecies with “gray-throated males” cluster into another. Overall, this result suggests that the two clades are independent lineages with strong speciation potential, since evolutionary forces can act rapidly on each isolated gene pool (Ellstrand & Elam, 1993; Wright, 1931). Meanwhile, the morphometric data show weak phenotypic differentiation between the two lineages (West-IT and East-IT), likely because they are lineages that have recently undergone diversification and have had insufficient time to develop a clear morphometric pattern. We found significant color differences among the three groups for most of the feather patches that we measured; the largest difference is in the throat patch. Finally, ecological data used to evaluate niche conservatism indicate that there are significant differences in the environmental spaces of the *D. baritula* groups: *baritula*, *montana* and *parva* groups.

### Well-supported divergence in *D. baritula* complex

Our phylogenomic tree, based on 178,160 SNPs, shows a split with 100% bootstrap support between the populations west of the Isthmus of Tehuantepec *versus* those from east of the isthmus. The short branches (Fig. S6) and limited phenotypic divergence (see following sections) suggest that this split was very recent, which is supported by our divergence time estimates (Fig. 2). It is known that the *D. baritula* complex originated during the Pleistocene, less than one million years ago (Barker et al., 2015, Fig. S1). By contrast, the SNAPP species tree suggested that *D. baritula* is not a monophyletic species (Fig. S5). Incomplete lineage sorting might explain the lack of reciprocal monophyly between *D. baritula* subspecies, as indicated in our ABBA-BABA test (Table 1). This pattern has been found in other young radiations, both in other genus *Diglossa* species (*carbonaria* complex; (Gutiérrez-Zuluaga et al., 2021)) and other bird groups such as Darwin’s finches (Freeland & Boag, 1999; Lamichhaney et al., 2015).

Since *D. baritula* is a resident species restricted to the Mesoamerican highlands, the Pleistocene climatic oscillations were likely a relevant factor in its current allopatric geographical distribution, as our results reveal (Figs. 2 and 5). During warmer interglacial periods, the highlands forests were reduced as they moved up to higher elevations (Hewitt, 2004; Barrantes, Iglesias & Fuchs, 2011). Consequently, the potential distribution of *D. baritula* was reduced and populations were isolated, facilitating genetic differentiation. Conversely, during cooler glacial periods, highlands vegetation descended to lower altitudes, which promoted their connectivity (Hewitt, 2004; Barrantes, Iglesias & Fuchs, 2011). This promoted geographic contact zones and gene flow events across *D. baritula* populations. The occurrence of two allopatric lineages in *D. baritula* provides evidence for its long-term persistence within separate refugia during Pleistocene glaciations. Such temporal and spatial population dynamics have impacted the patterns of genetic and phenotypic variation in our study species.

### The Isthmus of Tehuantepec as a biogeographical barrier

The Isthmus of Tehuantepec (IT) is the current topographic and ecological barrier between the West-IT Group and East-IT Group. The marked genetic divergence between populations (and subspecies) separated by the IT suggests that gene flow across this barrier has been reduced or interrupted owing to the low-elevation area of the IT. This geographic isolation is the major driver of intraspecific genetic divergence in *D. baritula*.

The IT is a lowland valley that appeared during the late Miocene (Barrier et al., 1998). This timing confirms again that the genetic divergence found here is a consequence of Pleistocene climatic oscillations, and not the emergence of the IT. The IT has played the role of an important geographic barrier in southern Mexico shaping the Mesoamerican highlands biodiversity, in birds (Pérez-Emán, 2005; Barber & Klicka, 2010; Zamudio-Beltrán et al., 2020) as well as other taxa such as reptiles (Bryson et al., 2011), mammals (León-Paniagua et al., 2007) and plants (Hernández-Langford, Siqueiros-Delgado & Ruíz-Sánchez, 2020).

### Partial congruence between phylogeny and phenotype (coloration and morphology)

Despite the phylogenetic inference of two well-supported clades in the *D. baritula* complex, we found weak phenotypic variation between them (Figs. 3 and 4). The two lineages observed here have diverged recently, leaving insufficient time for a clear phenotypic divergence pattern to emerge (Fig. 2). The West-IT Group and East-IT Group have not yet fully diverged and continue to share ancestral alleles due to incomplete lineage sorting (Table 1), which may explain the partial phenotypic split within *D. baritula*.

The coloration of the Cinnamon-bellied Flowerpiercer is consistent with the findings of Shultz & Burns (2017), who described differences in the evolution of female and male plumages corresponding to differences in their selective pressures. It is likely that the males’ ventral cinnamon plumage is a sexually selected trait, and thus the observed differences between subspecies might be due to the effect of diversifying selection. In birds, ventral patches are often involved in mate choice (Shultz & Burns, 2017; Merwin, Seeholzer & Smith, 2020). This suggests that different sexes and body parts are subjected to different intensities of sexual selection.

In our JNDs analyses we found significant color differences among the males of the three named subspecies for most feather patches we measured. These differences are likely to be biologically significant, since the magnitude of the difference is greater than the 1 JDN threshold for the colors to be perceived as different under the noise-constrained receptor visual model (Vorobyev & Osorio, 1998). The throat patch was the most strongly differentiated (*i.e.,* had the highest JND values). The most obviously diagnosable color trait was the difference in throat color between the West-IT and East-IT Group, *i.e.,* the two genomic lineages. In *D. b. baritula* the cinnamon ventral coloration extends up to the throat while in *D. b. montana* and *D. b. parva* the throat is gray. The sister group of the *D. baritul* a complex, *Diglossa plumbea*, is gray-throated, which could be the ancestral state for the group, with the cinnamon throat of *D. baritula* being a derived state. An ancestral state reconstruction including other related taxa would be of interest to clarify the evolution of color in these tanagers.

The throat patch coloration is more divergent between the subspecies isolated by the IT (cinnamon in *baritula vs.* gray in *montana* and *parva*) than between the *montana* and *parva* subspecies (both gray). Melanin is the most abundant pigment in bird feathers (Roulin et al., 2011) and consists of two types: eumelanin, which gives rise to black and gray colorations, and pheomelanin, which gives rise to yellowish to reddish colorations (McGraw, 2006; Galván & Solano, 2016). Both pigments are present in most melanic feathers (McGraw, 2004), but the dominant pigment type can be reliably assessed based on the color’s appearance and spectral curve (Galván & Wakamatsu, 2016). The West-IT Group throat color is predominantly due to pheomelanin pigmentation, while the East-IT Group the color is mainly eumelanin-derived. Different melanin ratios are implicated in pleiotropic associations providing fitness benefits under different selective conditions (Roulin et al., 2011; Roulin, 2015). Inter and intraspecific variation in melanin-based coloration is due to polymorphisms at the Mc1R gene (Roulin & Ducrest, 2013). Thus, a study with this gene would be helpful for solving the evolutionary history of *D. barirula*.

The small amount of variation found in the dorsal plumage of both males and females in *D. baritula* suggests that color in that body part might be constrained by their light environments. In many species with ‘drab’ melanin-based coloration, the light environment (*e.g.*, the light that filters through vegetation cover) is an important factor in determining plumage brightness, especially of dorsal patches (Marcondes & Brumfield, 2019). Moreover, their low-reflectance dorsal parts may decrease detection and aggression from conspecifics, competitors, and/or predators (Lyon & Montgomerie, 1986). In *D. baritula* males the dorsal part is gray, which is a eumelanin-based coloration, while the ventral part is cinnamon, which is a pheomelanin-based coloration. Thus, these results provide support for the idea that plumage evolution occurs in a patchwork fashion, with different parts of the plumage being subject to different selective pressures (Marcondes & Brumfield, 2019) as well as the hypothesis that ventral-dorsal variation is an important driver of sexual dichromatism (Marcondes & Brumfield, 2019).

In the morphological analyses, the first principal component (PC1) was most strongly related to bill length and bill hook length (BL and BHL), and moderately related to bill width (BW). Thus, we can consider PC1 to be a proxy for bill size. The bill is a highly variable character in our study species as well as in the genus *Diglossa* more generally (Mauck & Burns, 2009). The second principal component (PC2) was positively related to wing cord and tarsus length (WC and TL), which we considered to be a proxy of overall body size. In the scatterplot, we find a large degree of overlap between the *baritula* and *montana* groups, and weak differentiation between those two and *parva*. As in the individual measurement comparisons, this supports the trend of *parva* being smaller than the other two subspecies. These differences might indicate differences in thermal or feeding ecology (Mayr, 1956; Meiri & Dayan, 2003; Barnagaud et al., 2019). However, as the observed variation appears to be phylogenetically structured, it could be the result of isolation by distance, as has been observed in other highland bird taxa (Seeholzer & Brumfield, 2018).

Our results revealed that the only sexually dimorphic morphological trait is wing chord (WC), with males having longer WC than females. The wing is related to locomotion and flight performance in the environment (Tobias et al., 2022). The longer wings of males decrease the flight energy required in courtship display (Møller, 1991; Sun et al., 2017). However, it may not only be by the force of sexual selection solely but might also have an ecological cause. For example, if females are more vulnerable to predation when they are incubating eggs, shorter wings may improve their maneuverability and help them evade predators (Swaddle & Lockwood, 2003; Bomberger & Brown, 2011). If we assume that wing length is a good indicator of body size (Ashton, 2002), we provide the first evidence for sexual dimorphism in body size in *D. baritula*. This pattern of differences between sexes in wing chord have been reported in other birds (Robles-Bello et al., 2022; Thom et al., 2008; Sun et al., 2017).

Within the East-IT Group, we found geographical differentiation for bill length (BL) in the form of clinal variation from large BL in *D. b. montana* to small BL in *D. b. parva*. Since geographic variation refers to intraspecific phenotypic differentiation (Mayr, 1969) and the bill is related to foraging (Tobias et al., 2022), populations of *D. b. montana* and *D. b. parva* might be adapted to their specific local diets. Another example of geographic variation within the East-IT Group could be total length. Although we did not measure total length, photographs of skins show that *D. b. parva* has a smaller total length than *D. b. montana* (Fig. 1), this variation could be evidence of local thermal adaptation (Friedman & Remeš, 2017).

The tarsus length (TL) is congruent with the phylogenetic inference; the East-IT Group has a shorter TL than the West-IT Group. TL is generally used as a proxy of body size (Zink & Remsen, 1986; Yom-Tov, 2001; Töpfer, 2018). Since intraspecific variation in body size represents a thermoregulatory adaptation to local environmental niches (Mayr, 1956; Pigot et al., 2020) and the *D. baritula* complex is sedentary, its populations must adapt to their habitats and are likely to follow patterns such as Bergmann’s (1847) rule, which describes the tendency of animals to have larger individuals in higher latitudes (Graves, 1991) or in cooler areas, given that climate is correlated with latitude (Ashton, 2002). Therefore, we propose that vicariance plays an important role in the *D. baritula* complex. The trend of smaller individuals to the east than to the west of the IT has been found in other birds (*e.g.*, Rodríguez-Gómez et al., 2021).

### Differences in ecological niche within the *D. baritula* complex

The Pleistocene climatic oscillations played an important role in the cinnamon-bellied flowerpiercer evolutionary history. Its movements to suitable habitats that fulfill its biogeographic affinities and ecological requirements determined the response of *D. baritula* to the glacial and interglacial periods. It is well-known that historical processes, such as Pleistocene climatic oscillations, have played an important role in shaping current biodiversity (*e.g.*, Castillo-Chora et al., 2021). The Pleistocene was a geological period with severe global alternations between glacial and interglacial cycles (Hewitt, 2004; Ramírez-Barahona & Eguiarte, 2013).

Ecological niche differentiation, one of the drivers of lineage divergence, was confirmed by the niche overlap tests, which suggest that the three subspecies have different potential environmental spaces (Fig. 6). We interpreted our results of the equivalency and similarity tests mainly based on the significance values and the low observed D values. Equivalency tests performed to compare the observed and expected niche spaces revealed that the niches are not equivalent. In background similarity tests, we found that pairs of realized subspecies niches were more different than expected by chance as they occupy areas with different environmental conditions (Fig. 6). Differentiation in niches may indicate that each subspecies has different ecological tolerances and their own distribution range (Warren, Glor & Turelli, 2008). Thus, climatic variables have played an important role in promoting adaptive divergence within the *D. baritula* complex. Differences in environmental spaces among subspecies are explained by the differences in climatic conditions where they inhabit. While *D. b. baritula* occupy regions with less amount of rainfall, for example pine-oak forests, *D. b. montana* and *D. b. parva* inhabit montane regions with marked seasonality (in both temperature and precipitation), for example cloud forests (Helmer et al., 2019; Ruiz-Sanchez & Ornelas, 2014; Ortiz-Rodriguez, Ornelas & Ruiz-Sánchez, 2018).

## Conclusion

Within the *D. baritula* complex, current taxonomic limits (*i.e.,* subspecies) do not correspond to the genomic and phenotypic splits presented in this study. We detected two evolutionarily independent genomic units: one includes only *D. b. baritula,* while the other contains both the *D. b. montana* and *D. b. parva* subspecies. In contrast to the genomic results, phenotypic data showed intermediate levels of differentiation, indicating that population groups within *D. baritula* have probably been under similar evolutionary pressures, perhaps natural selection maintains uniform phenotypes despite genomic differentiation. On the other hand, ecological data revealed that each subspecies occupies different environmental spaces, suggesting that environmental factors have influenced the evolution within this complex.

This is the first study at the genomic, phenetic and ecological levels within *D. baritula* to include samples of all three subspecies. Our data demonstrated that there is intraspecific genetic divergence, which might suggest that *D. baritula* is made up of two different species. Alternatively, the cinnamon-bellied flowerpiercer is in the process of incipient speciation, a “gray zone” of speciation (de Queiroz, 2007) where genetic variation occurs before notable phenotypic variation has developed and both lineages fall on the speciation continuum (Shaw & Mullen, 2014; Stankowski & Ravinet, 2021). Making inferences about species limits is challenging taking into account the genomic sampling sizes used here, and the cryptic phenotypes that have not completely diverged. More extensive genomic sampling is needed to fully resolve the taxonomic status of the two lineages.

In particular, the present investigation illustrates the evolutionary potential of the *D. baritula* complex, and in general, highlights the capacity of the Mesoamerican highlands to create cryptic biodiversity and endemism. Climatic fluctuations during the Pleistocene facilitated the diversification and distribution in our study group. Hence, the Mesoamerican highlands might act as future refugia for species faced with the present climate change, and we emphasize the necessity of their conservation. Moreover, recognition of the different genetic stocks is a useful basis to recognize and treat differentiated lineages as separate units for conservation management.

##  Supplemental Information

10.7717/peerj.16797/supp-1 Supplemental Information 1 Supplemental Figures

## Additional Information and Declarations

### DNA Deposition

The following information was supplied regarding the deposition of DNA sequences: The data is available at GenBank, PRJNA901463, and at figshare: Terrones Ramirez, Alondra K. (2022). fastq_baritula.tar. figshare. Dataset. https://doi.org/10.6084/m9.figshare.21540072.v1.

### Data Availability

The following information was supplied regarding data availability: The color, morphometrics and ecological niche modeling data are available at Zenodo: AloTerrones. (2023). AloTerrones/Terrones-Ramirez_et_al_2023_D_baritula: Terrones-Ramirez_D_baritula_2023 (v1.0.0). Zenodo. https://doi.org/10.5281/zenodo.8019104.
