# Supplementary material for: Recent genetic, phenetic and ecological divergence across the Mesoamerican highlands: a study case with Diglossa baritula (Aves: Thraupidae)
Source: PeerJ. 2024 Mar 22;12:e16797. doi: 10.7717/peerj.16797 (PMC10962342; doi:10.7717/peerj.16797)
Supplement: Supplemental Information 1 [file peerj-12-16797-s001.pdf]

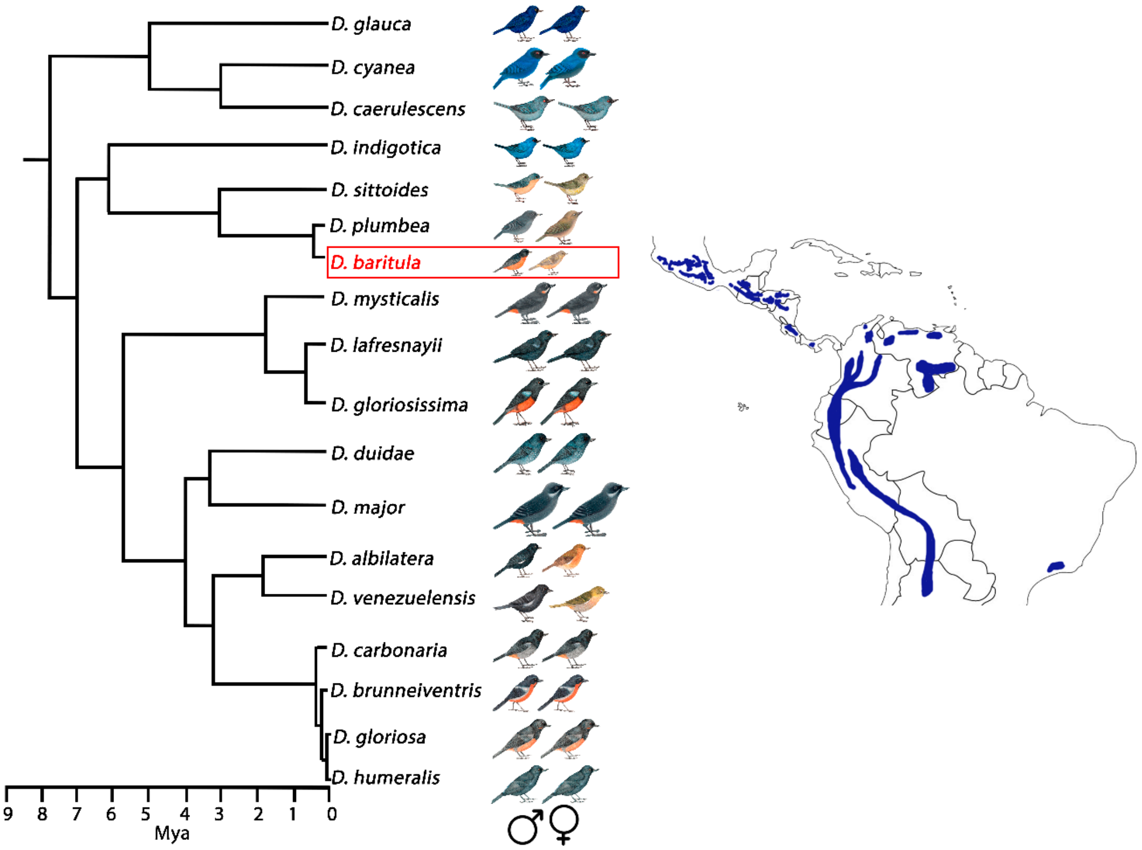

Figure S1. Evolutionary radiation of the *Diglossa* genus, showing that in less than 10 million years there has been a great diversification in patterns of coloration, body size, etc. Dated phylogeny modified from Barker et al., 2015, in red the species of this study. The illustrations of the females and males were made by Arantza Lujambio-Ramírez, J. Alberto Hernández-Martínez and Margarita I. Reyes-Vargas.

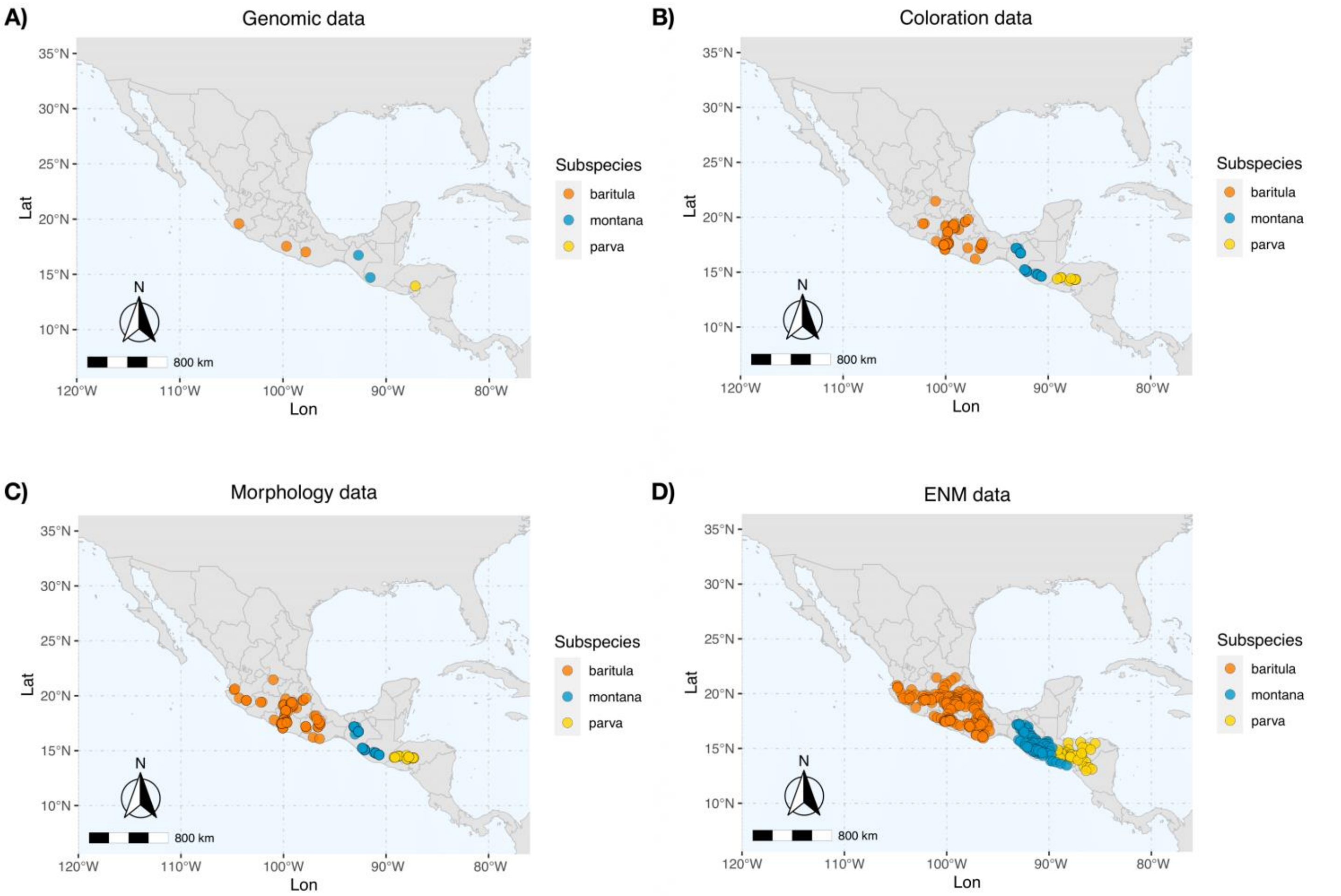

Figure S2. Distribution of samples used in this study. A) Samples used for genomic analysis, n=6. B) Data used in coloration analysis, n=85. C) Data used in morphological analysis, n=75. D) Occurrence data used in Ecological Niche Modeling analysis, n=288. Subspecies distribution is shown in different colors: *D. b. baritula* in orange, *D. b. montana* in blue and *D. b. parva* in yellow.

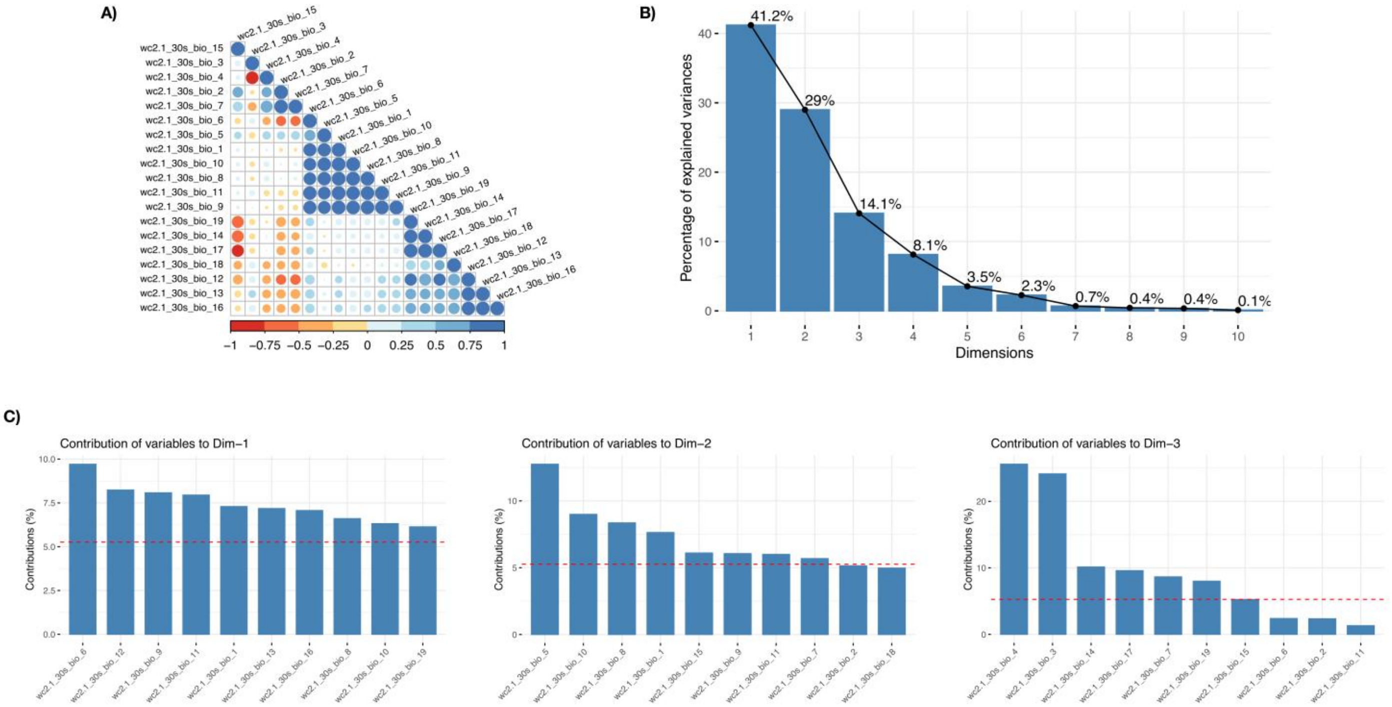

Figure S3. Correlation and Principal Component Analysis taken into account for the selection of bioclimatic variables in Ecological Niche modeling analysis. A) Pearson correlation (*r*>0.075); B) Percentage of explained variance for the first 10 principal components; C) Contribution of variables in the first three principal components. These analyses were performed using the 288 occurrence points for the *D. baritula* complex.

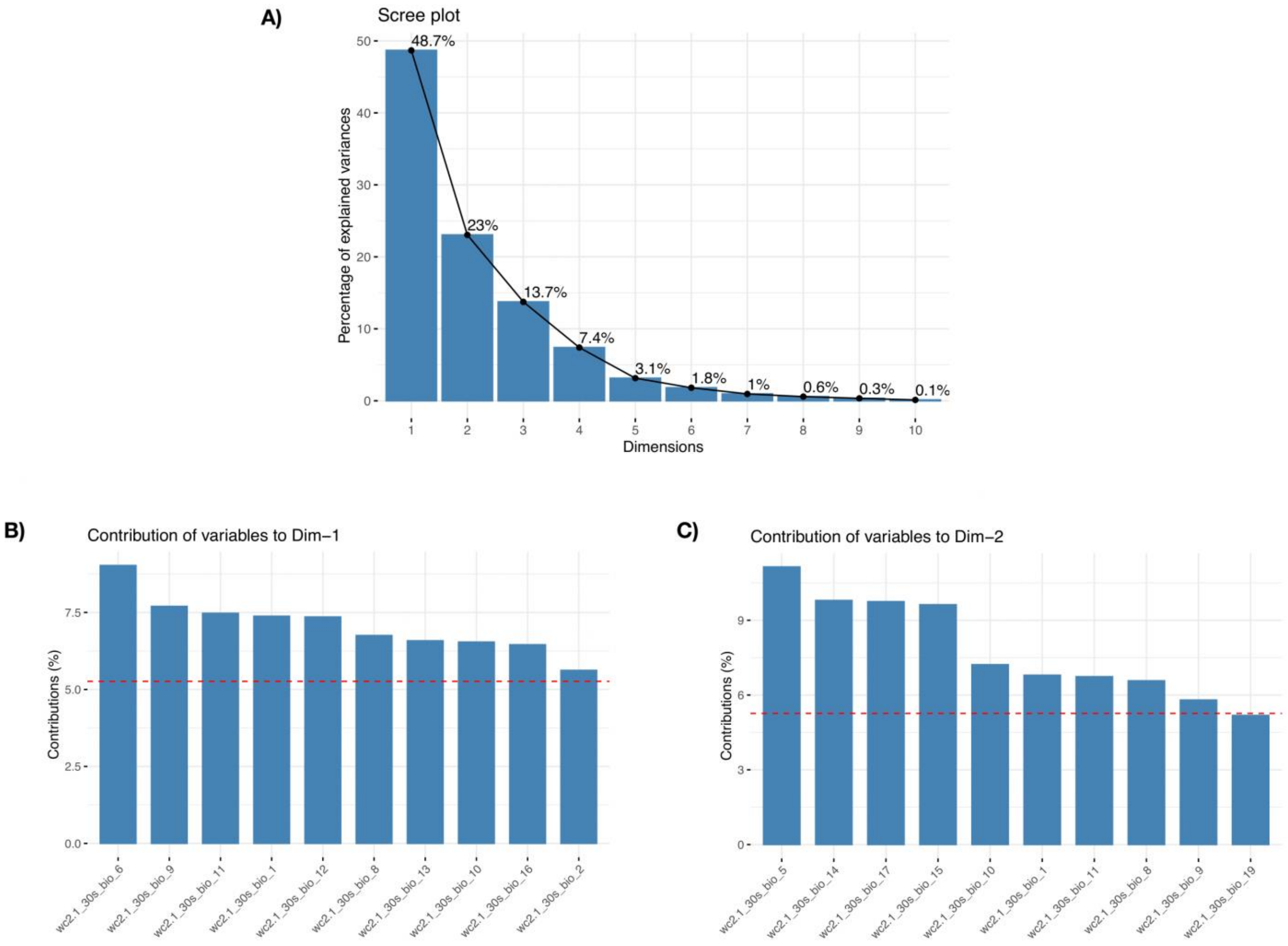

Figure S4. Principal Component Analysis performed in Niche Overlap analysis. A) Percentage of explained variance for the first 10 principal component analysis; B) Contribution of variables for the first principal components; C) Contribution of variables for the first principal components. These analyses were performed using the 288 occurrence points for the *D. baritula* complex, and the background points for the accessible area for the species.

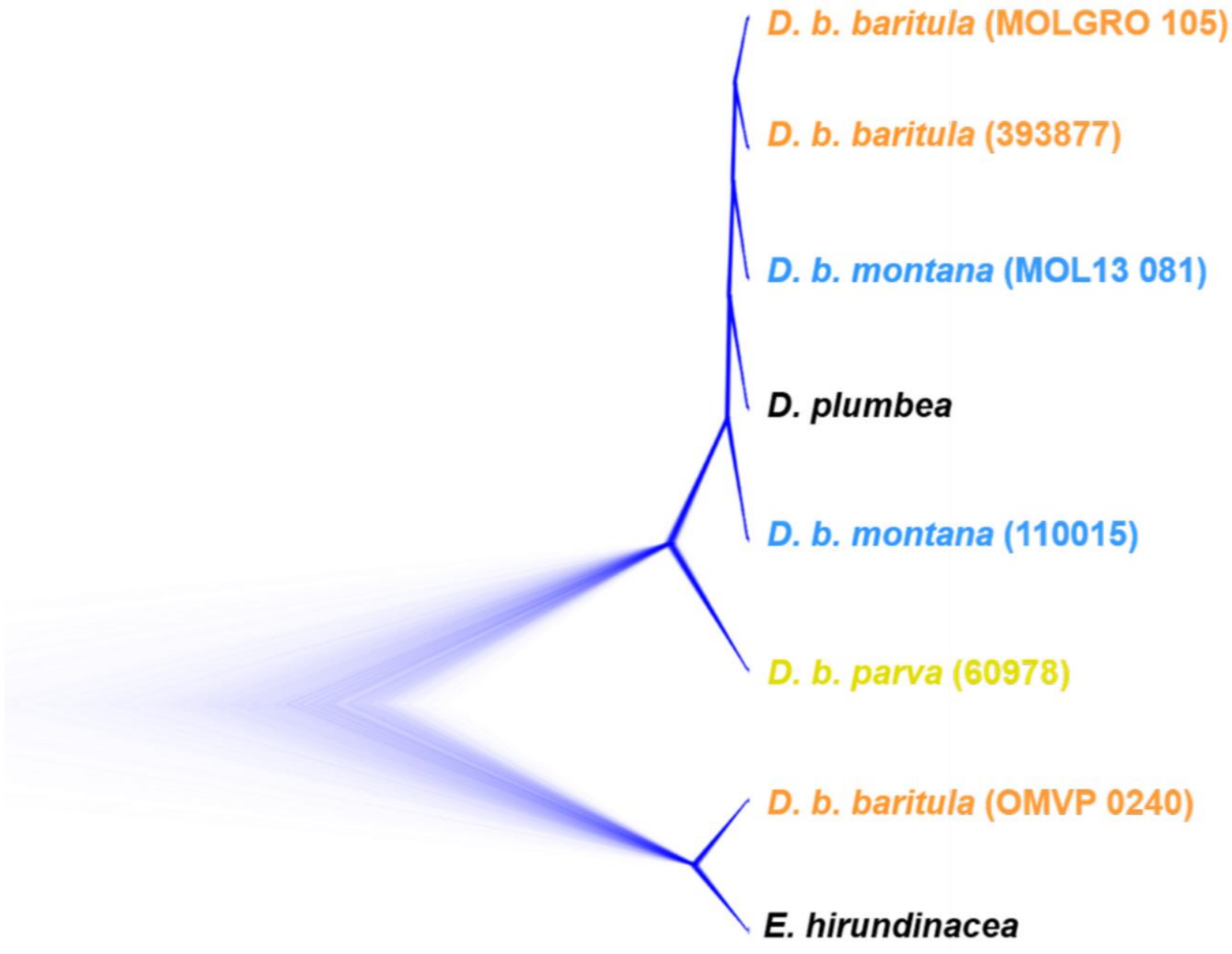

Figure S5. SNAPP species tree obtained from 33,702 loci RADseq loci. In orange *D. b. baritula*, in blue *D. b. montana* and in yellow *D. b. parva*.

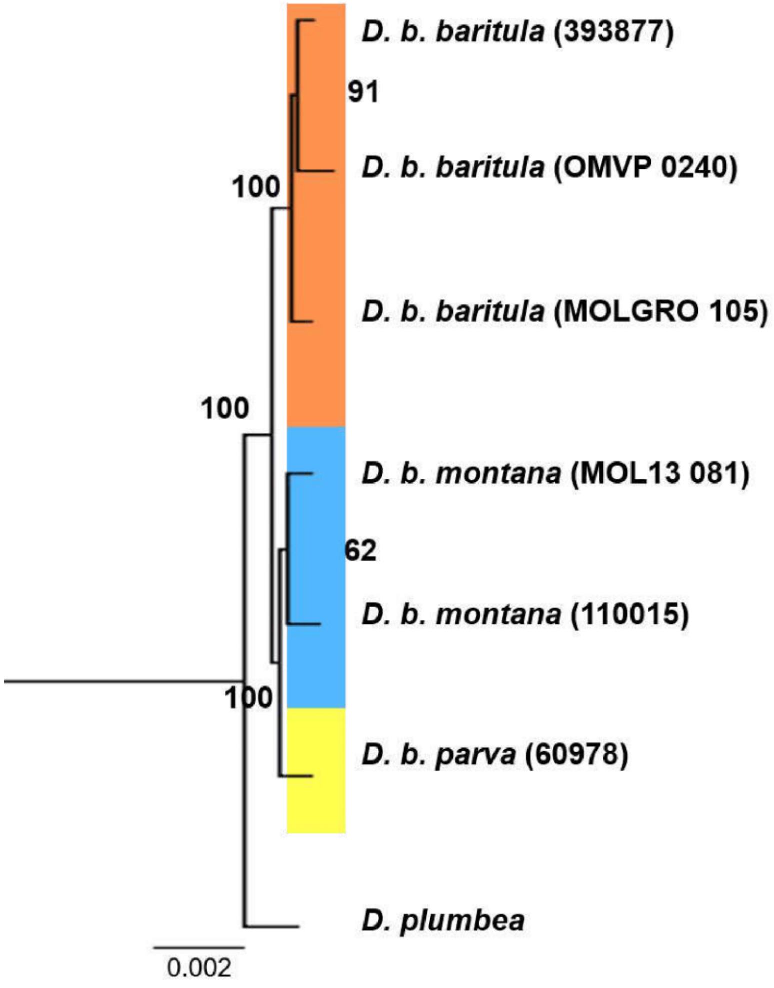

Figure S6. Maximum likelihood phylogenomic tree obtained from 43,704 RADseq loci representing phylogenetic relationships among clades in the *Diglossa baritula* complex, in orange *D. b. baritula*, in blue *D. b. montana* and in yellow *D. b. parva*. Numbers indicate bootstrap node supports. *Diglossa plumbea* and *Euphonia hirundinacea* are used as sister group and outgroup, respectively.

Table S1 Taxonomic proposals for *Diglossa* species.

Reference	Taxonomic hypothesis	Data used
Cassin (1864) & Sclater (1875)	The species were included in two genera, *Diglossa* and *Diglossopis*, the latter being a monotypic genus containing only *Diglossopis* caerulescens, which has the smallest bill.	Bill size and coloration
Hellmayr (1935)	Some closely related species were combined, reducing the number of species.	Morphology
Vuilleumier (1969)	Two ranges of taxonomic classification were proposed: four "species groups" (*major*, *lafresnayii*, *albilatera* and *caerulescens*) and four "superspecies" (*lafresnayii*, *carbonaria*, *baritula*, and *albilatera*).	Bill and tongue morphology, coloration
Bock (1985)	The genus *Diglossopis* was returned, where *D. caerulescens*, *D. cyanea* and *D. glauca* were included.	Skull, tongue and bill morphology
Sibley & Monroe (1990)	*D. indigotica* was moved to *Diglossopis*.	DNA-DNA hybridization
Dickinson (2003) and Remsen et al. (2008)	*Diglossopis* was absorbed by *Diglossa*.	Not specified

Table S2 Taxonomic positions for the *D. baritula* complex.

Reference	Taxonomic classification
Wagler (1832)	*D. baritula* species was named, which is the type species of the genus.
Dearbon (1907)	*D. b. montana* subspecies was described.
Griscom (1932)	The third subspecies, *D. b. parva*, was named
Hellmayr (1935)	*D. baritula*, *D. plumbea* and *D. sittoides*grouped as a single species.
Friedmann et al. (1950)	Two subspecies were reported: *D. b. baritula* (pico chueco mexicano) and *D. b. montana* (pico chueco chiapaneco).
Skutch (1954)	*D. baritula* and *D. plumbea* combined as a single species with three subspecies: *D. b. baritula*, *D. b. montana* and *D. b. plumbe.*
Vuilleumier (1969)	*D. baritula* was grouped with *D. montana* and *D. sittoides* in the *baritula* superspecies.
Monroe (1968) and Isler & Isler (1987)	Three subspecies were mentioned: *D. b. baritula*, *D. b. montana* and *D. b. parva.*

Table S3 Information for the *Diglossa baritula* specimens included in this study.

Subspecies	Coll	ID sample	G	Lat	Long	Locality	Gen	Morp	Col
D. b. baritula	CNAV	11045	F	19.8	-104.32333	Mexico, Jalisco, Reserva de la Biosfera de Miahuatlan		X	
D. b. baritula	CNAV	11047	F	18.15	-99.9	Mexico, Guerrero, Tepoxtepec		X	
D. b. baritula	CNAV	11048	F	17.41666	-100.11666	Mexico, Guerrero, Atoyac de Álvarez		X	
D. b. baritula	CNAV	11050	F	17.01666	-97.75	Mexico, Oaxaca, Santiago Nuyoo		X	
D. b. baritula	CNAV	11054	M	19.26166	-99.99166	Mexico, Mexico state, Amanalco		X	X
D. b. baritula	CNAV	11055	F	19.706934	-99.786552	Mexico, Mexico state, Jocotitlán		X	
D. b. baritula	CNAV	11057	F	19.11666	-100.01666	Mexico, Mexico state, Temascaltepec		X	
D. b. baritula	CNAV	11058	M	19.11666	-100.01666	Mexico, Mexico state, Temascaltepec		X	X
D. b. baritula	CNAV	11059	F	19.29527	-99.24	Mexico, Mexico city, La Magdalena Contreras		X	
D. b. baritula	CNAV	11060	M	19.29527	-99.24	Mexico, Mexico city, La Magdalena Contreras		X	X
D. b. baritula	CNAV	11061	M	19.29527	-99.24	Mexico, Mexico city, La Magdalena Contreras		X	X
D. b. baritula	CNAV	11062	M	19.6	-98.08333	Mexico, Tlaxcala, Tlaxco		X	X
D. b. baritula	CNAV	11063	M	19.6	-98.08333	Mexico, Tlaxcala, Tlaxco		X	X
D. b. baritula	CNAV	11064	M	19.6	-98.08333	Mexico, Tlaxcala, Tlaxco		X	X
D. b. baritula	CNAV	11065	M	18.89166	-98.72833	Mexico, Morelos, Tetela		X	X
D. b. baritula	CNAV	11066	M	19.01666	-99.09333	Mexico, Morelos, Tetela		X	X
D. b. baritula	CNAV	14180	F	19.16833	-99.90333	Mexico, Morelos, Tetela		X	
D. b. baritula	CNAV	14687	M	16.21666	-97.11666	Mexico, Oaxaca, La Cima		X	X
D. b. baritula	CNAV	14688	M	18.15	-99.9	Mexico, Guerrero, Tepoxtepec		X	X
D. b. baritula	CNAV	14689	M	17.16666	-96.61666	Mexico, Oaxaca, Cerro San Felipe		X	X
D. b. baritula	CNAV	14690	M	17.16333	-96.675	Mexico, Oaxaca, Cerro San Felipe		X	X
D. b. baritula	CNAV	14719	F	17.16666	-96.61666	Mexico, Oaxaca, Cerro San Felipe		X	
D. b. baritula	CNAV	17555	M	19.03333	-99.205	Mexico, Morelos, Coajomulco		X	X
D. b. baritula	CNAV	22951	M	19.2647	-99.299	Mexico, Mexico city, Dinamo 4		X	X
D. b. baritula	CNAV	22952	M	19.2647	-99.299	Mexico, Mexico city, Dinamo 4		X	X
D. b. baritula	CNAV	24849	M	17.3970306	-96.428366	Mexico, Oaxaca, Cerro de los Pozuelos		X	X
D. b. baritula	CNAV	24850	F	17.3970306	-96.428366	Mexico, Oaxaca, Cerro de los Pozuelos		X	
D. b. baritula	CNAV	27132	M	19.42241	-102.088	Mexico, Michoacán, Parque Nacional del Cupatitzio		X	X
D. b. baritula	AMNH	40345	M	19.4326009	-99.1333416	Mexico, Mexico city		X	X
D. b. baritula	AMNH	105891	M	19.579	-103.623	Mexico, Jalisco, Volcán de Nieve		X	
D. b. baritula	AMNH	105892	M	19.579	-103.623	Mexico, Jalisco, Volcán de Nieve		X	
D. b. baritula	AMNH	105893	F	19.579	-103.623	Mexico, Jalisco, Volcán de Nieve		X	
D. b. baritula	FMNH	393877	ND	19.591827	-104.265508	Mexico, Jalisco, Las Joyas	X		
D. b. baritula	AMNH	508149	M	20.537108	-104.81302	Mexico, Jalisco, Mascota		X	
D. b. baritula	AMNH	508150	M	20.628395	-104.739202	Mexico, Jalisco, Juanacatlán		X	
D. b. baritula	AMNH	778545	M	19.018976	-99.26216	Mexico, Morelos, Cuernavaca		X	X
D. b. baritula	MZFC	AGH 032	M	17.065	-100.0666667	Mexico, Guerrero, Omiltemi		X	X
D. b. baritula	MZFC	AGH SN 5025	M	17.065	-100.0666667	Mexico, Guerrero, Omiltemi		X	X
D. b. baritula	MZFC	AGNS 0175	M	19.295	-99.24	Mexico, Mexico city, Primer Dinamo		X	X
D. b. baritula	MZFC	AGNS 0206	M	17.5	-100.2666667	Mexico, Guerrero, Toro Muerto		X	X
D. b. baritula	MZFC	AGNS 0216	F	17.5	-100.2666667	Mexico, Guerrero, Toro Muerto		X	
D. b. baritula	MZFC	AGNS 0366	M	17.4833333	-100.2	Mexico, Guerrero, El Iris		X	X
D. b. baritula	MZFC	AGNS 1023	F	17.515	-96.505	Mexico, Oaxaca, La Esperanza		X	
D. b. baritula	MZFC	AGNS 1024	M	17.51	-96.50333333	Mexico, Oaxaca, La Esperanza		X	X
D. b. baritula	MZFC	AGNS SN 02391	F	19.295	-99.24	Mexico, Mexico city, Primer Dinamo		X	
D. b. baritula	MZFC	AGNS SN 03996	F	17.4833333	-100.2	Mexico, Guerrero, El Iris		X	
D. b. baritula	MZFC	AGNS SN 04983	F	17.5	-100.2666667	Mexico, Guerrero, El Iris		X	

D. b. baritula	MZFC	AGNS SN 05026	M	17.065	-100.0666667	Mexico, Guerrero, Omiltemi		X	X
D. b. baritula	MZFC	AGNS SN 05027	M	17.7416666	-99.72666667	Mexico, Guerrero, Omiltemi		X	X
D. b. baritula	MZFC	AGNS SN 05030	F	17.7416666	-99.72666667	Mexico, Guerrero, Omiltemi		X	
D. b. baritula	MZFC	AGNS SN 05032	F	17.065	-100.0666667	Mexico, Guerrero, Omiltemi		X	
D. b. baritula	MZFC	AGNS SN 05034	F	17.065	-100.0666667	Mexico, Guerrero, Omiltemi		X	
D. b. baritula	MZFC	AHC 099	M	19.79948	-97.80345	Mexico, Puebla, Chopilco Alto		X	X
D. b. baritula	MZFC	ATP2002 18	F	19.25715	-99.03473333	Mexico, Mexico city, Ajusco medio	*		
D. b. baritula	MZFC	BEHB 009	M	17.705	-96.4116666	Mexico, Oaxaca, Puerto Eligio		X	X
D. b. baritula	MZFC	BIODF 060	F	19.2182493	-99.06302778	Mexico, Mexico city, Axomulco		X	
D. b. baritula	MZFC	CONACYT 770	F	18.165	-96.99666667	Mexico, Oaxaca, Puerto de la Soledad		X	
D. b. baritula	MZFC	DONAD 059	F	19.3901944	-99.20422222	Mexico, Mexico city, UNAM		X	
D. b. baritula	MZFC	GUER 17	M	17.61583	-99.83861	Mexico, Guerrero, Carrizal de Bravo		X	X
D. b. baritula	MZFC	GUER 18	F	17.61583	-99.83861	Mexico, Guerrero, Carrizal de Bravo		X	
D. b. baritula	MZFC	IFFD 0018	M	17.8074167	-100.9154667	Mexico, Guerrero, Los Vergeles		X	X
D. b. baritula	MZFC	INECOL 073	F	17.2054056	-97.83656111	Mexico, Oaxaca, Chicahuaxtla		X	
D. b. baritula	MZFC	INECOL 074	F	17.2054056	-97.83656111	Mexico, Oaxaca, Chicahuaxtla		X	
D. b. baritula	MZFC	INECOL 075	M	17.2054056	-97.83656111	Mexico, Oaxaca, Chicahuaxtla		X	X
D. b. baritula	MZFC	JEMP 360	F	18.566	-99.6	Mexico, Guerrero, El Huizteco		X	
D. b. baritula	MZFC	JEMP 503	F	18.65	-99.78333333	Mexico, Guerrero, Los Jarillos		X	
D. b. baritula	MZFC	JEMP 505	M	18.65	-99.78333333	Mexico, Guerrero, Los Jarillos		X	X
D. b. baritula	MZFC	JEMP 506	M	18.65	-99.78333333	Mexico, Guerrero, Los Jarillos		X	X
D. b. baritula	MZFC	JEMP 507	M	18.65	-99.78333333	Mexico, Guerrero, Los Jarillos		X	X
D. b. baritula	MZFC	JK04 111	F	17.816713	-99.967595	Mexico, Guerrero, Carrizal de Bravo		X	
D. b. baritula	MZFC	JK04 112	F	17.816713	-99.967595	Mexico, Guerrero, Carrizal de Bravo		X	
D. b. baritula	MZFC	JK04 164	M	17.816713	-99.967595	Mexico, Guerrero, Carrizal de Bravo		X	X
D. b. baritula	MZFC	JK04 174	F	17.816713	-99.967595	Mexico, Guerrero, Carrizal de Bravo		X	
D. b. baritula	MZFC	JK04 176	F	17.816713	-99.967595	Mexico, Guerrero, Carrizal de Bravo		X	
D. b. baritula	CNAV	JK11 201	M	17.5741	-99.69	Mexico, Guerrero, Omiltemi		X	X
D. b. baritula	CNAV	JK11 206	M	17.5741	-99.69	Mexico, Guerrero, Omiltemi		X	X
D. b. baritula	CNAV	JMD 437	F	17.614166	-99.851	Mexico, Guerrero, Leonardo Bravo		X	
D. b. baritula	CNAV	LGM 08	M	21.46262	-100.99472	Mexico, Guanajuato, San Diego de la Unión		X	X
D. b. baritula	CNAV	MM 458	M	19.4231644	-102.233	Mexico, Michoacán, Nuevo Parangaricutiro		X	X
D. b. baritula	MZFC	MOL15 31	F	16.08972	-96.48548	Mexico, Oaxaca, Puente Río Molino		X	
D. b. baritula	MZFC	MOLGRO 105	M	17.55	-99.66666667	Mexico, Guerrero, Omiltemi	X		
D. b. baritula	MZFC	MOLGRO 143	F	17.55	-99.66666667	Mexico, Guerrero, Omiltemi		X	
D. b. baritula	MZFC	MOLGRO 150	F	17.55	-99.66666667	Mexico, Guerrero, Omiltemi		X	
D. b. baritula	MZFC	MOLGRO 193	M	17.58668	-99.83707	Mexico, Guerrero, Carrizal de Bravo		X	X
D. b. baritula	MZFC	MOLGRO 242	M	17.58668	-99.83707	Mexico, Guerrero, Carrizal de Bravo		X	X
D. b. baritula	MZFC	MOLGRO 243	F	17.58668	-99.83707	Mexico, Guerrero, Carrizal de Bravo		X	
D. b. baritula	MZFC	MOLGRO 244	F	17.58668	-99.83707	Mexico, Guerrero, Carrizal de Bravo		X	
D. b. baritula	MZFC	MOLGRO 245	M	17.58668	-99.83707	Mexico, Guerrero, Carrizal de Bravo		X	X
D. b. baritula	MZFC	MT 157	M	17.515	-96.505	Mexico, Oaxaca, La Esperanza		X	X
D. b. baritula	MZFC	OMVP 0090	F	18.17	-96.84666667	Mexico, Oaxaca, Sierra de Huautla		X	
D. b. baritula	MZFC	OMVP 0240	ND	17.025	-97.795	Mexico, Oaxaca, Santa María Yucuhiti	X		
D. b. baritula	MZFC	OMVP 0972	F	17.845	-96.74	Mexico, Oaxaca, Peña Verde		X	
D. b. baritula	MZFC	PEP 352	M	17.4666667	-100.1666667	Mexico, Guerrero, Puerto Gallo		X	X
D. b. baritula	MZFC	PEP 364	M	17.4666667	-100.1666667	Mexico, Guerrero, Puerto Gallo		X	X
D. b. baritula	MZFC	PEP 368	M	17.4666667	-100.1666667	Mexico, Guerrero, Puerto Gallo		X	X
D. b. baritula	MZFC	PEP 371	F	17.4666667	-100.1666667	Mexico, Guerrero, Puerto Gallo		X	
D. b. baritula	MZFC	PEP 396	F	17.4666667	-100.1666667	Mexico, Guerrero, Puerto Gallo		X	
D. b. baritula	MZFC	PEP 602	M	17.4666667	-100.1666667	Mexico, Guerrero, Puerto Gallo		X	X
D. b. baritula	MZFC	PEP 762	F	17.55	-99.66666667	Mexico, Guerrero, Omiltemi		X	

D. b. montana	USNM	30724	M	14.8	-91.0167	Guatemala, Chimaltenango, Tecpán		X	X
D. b. montana	UWBM	110015	ND	14.7166667	-91.535	Guatemala, Quetzaltenango, Santa María de Jesús	X		
D. b. montana	USNM	349723	M	14.86666	-91.21666	Guatemala, Totonicapán, María Tecun		X	X
D. b. montana	USNM	349724	M	14.8	-91.0167	Guatemala, Chimaltenango, Tecpán		X	X
D. b. montana	USNM	349725	F	14.8	-91.0167	Guatemala, Chimaltenango, Tecpán		X	
D. b. montana	AMNH	398054	M	14.76667	-91	Guatemala, Chimaltenango, Tecpán		X	X
D. b. montana	AMNH	398055	M	14.76667	-91	Guatemala, Chimaltenango, Tecpán		X	
D. b. montana	AMNH	398056	F	14.76667	-91	Guatemala, Chimaltenango, Tecpán		X	
D. b. montana	AMNH	398057	F	14.8	-91.0167	Guatemala, Chimaltenango, Tecpán		X	
D. b. montana	AMNH	398062	M	14.61667	-90.66667	Guatemala, Sacatepéquez, San Lucas		X	X
D. b. montana	AMNH	398063	M	14.61667	-90.66667	Guatemala, Sacatepéquez, San Lucas		X	X
D. b. montana	AMNH	398064	M	14.61667	-90.66667	Guatemala, Sacatepéquez, San Lucas		X	X
D. b. montana	AMNH	398065	F	14.61667	-90.66667	Guatemala, Sacatepéquez, San Lucas		X	
D. b. montana	AMNH	748477	M	17.1599	-92.8999	Mexico, Chiapas, Pueblo Nuevo Solistahuacán		X	X
D. b. montana	MZFC	BEHB08 14	F	16.7278528	-92.69638889	Mexico, Chiapas, Cerro Huitepec		X	
D. b. montana	MZFC	BEHB08 26	F	16.7278528	-92.69638889	Mexico, Chiapas, Cerro Huitepec		X	
D. b. montana	MZFC	BEHB08 38	M	16.7278528	-92.69638889	Mexico, Chiapas, Cerro Huitepec		X	X
D. b. montana	MZFC	BEHB08 46	M	16.7278528	-92.69638889	Mexico, Chiapas, Cerro Huitepec		X	X
D. b. montana	MZFC	BMM 866	M	15.1316667	-92.10833333	Mexico, Chiapas, Volcán Tacaná		X	X
D. b. montana	MZFC	BMM 879	F	15.1316667	-92.10833333	Mexico, Chiapas, Volcán Tacaná		X	
D. b. montana	MZFC	BONA 37	M	15.0666667	-92.08333333	Mexico, Chiapas, Volcán Tacaná		X	X
D. b. montana	MZFC	BONA 50	M	15.0666667	-92.08333333	Mexico, Chiapas, Volcán Tacaná		X	X
D. b. montana	MZFC	BONA 70	F	15.0666667	-92.08333333	Mexico, Chiapas, Volcán Tacaná		X	
D. b. montana	MZFC	BONA 71	M	15.0666667	-92.08333333	Mexico, Chiapas, Volcán Tacaná		X	X
D. b. montana	MZFC	BONA 73	F	15.0666667	-92.08333333	Mexico, Chiapas, Volcán Tacaná		X	
D. b. montana	MZFC	BONA 86	F	15.0666667	-92.08333333	Mexico, Chiapas, Volcán Tacaná		X	
D. b. montana	CNAV	BRB 782	F	16.5026	-93.0193	Mexico, Chiapas, Chiapa de Corzo		X	
D. b. montana	MZFC	CRGA 24	F	15.23541	-92.30463	Mexico, Chiapas, Cerro Boquerón		X	
D. b. montana	MZFC	CRGA 45	F	15.23541	-92.30463	Mexico, Chiapas, Cerro Boquerón		X	
D. b. montana	MZFC	CRH 024	F	17.16613	-93.14163	Mexico, Chiapas, Coapilla		X	
D. b. montana	MZFC	CRH 031	M	17.16613	-93.14163	Mexico, Chiapas, Coapilla		X	X
D. b. montana	MZFC	EAGT 830	M	15.23541	-92.30463	Mexico, Chiapas, Cerro Boquerón		X	X
D. b. montana	MZFC	EAGT 841	M	15.23541	-92.30463	Mexico, Chiapas, Cerro Boquerón		X	X
D. b. montana	MZFC	MFOR 736	M	17.18758	-93.12125	Mexico, Chiapas, Tapalapa		X	X
D. b. montana	MZFC	MFOR 744	M	17.18758	-93.12125	Mexico, Chiapas, Tapalapa		X	X
D. b. montana	MZFC	MOL13 008	F	16.7380556	-92.68805556	Mexico, Chiapas, Cerro Huitepec		X	
D. b. montana	MZFC	MOL13 014	F	16.7380556	-92.68805556	Mexico, Chiapas, Cerro Huitepec		X	
D. b. montana	MZFC	MOL13 037	M	16.7380556	-92.68805556	Mexico, Chiapas, Cerro Huitepec		X	X
D. b. montana	MZFC	MOL13 038	M	16.7380556	-92.68805556	Mexico, Chiapas, Cerro Huitepec		X	X
D. b. montana	MZFC	MOL13 071	M	16.7380556	-92.68805556	Mexico, Chiapas, Cerro Huitepec		X	X
D. b. montana	MZFC	MOL13 080	F	16.7380556	-92.68805556	Mexico, Chiapas, Cerro Huitepec		X	
D. b. montana	MZFC	MOL13 081	M	16.7380556	-92.68805556	Mexico, Chiapas, Cerro Huitepec	X	X	X
D. b. montana	MZFC	MOL13 083	F	16.7380556	-92.68805556	Mexico, Chiapas, Cerro Huitepec		X	
D. b. montana	MZFC	MOL13 093	F	16.7380556	-92.68805556	Mexico, Chiapas, Cerro Huitepec		X	
D. b. montana	MZFC	MOL13 131	M	16.7380556	-92.68805556	Mexico, Chiapas, Cerro Huitepec		X	X
D. b. parva	MLZ	16844	F	14.4	-89.23333	Honduras, Ocotepeque, El Chorro		X	
D. b. parva	MLZ	18353	F	14.516667	-88.75	Honduras, Ocotepeque, Cerro Verde		X	
D. b. parva	MLZ	18355	F	14.516667	-88.75	Honduras, Ocotepeque, Cerro Verde		X	
D. b. parva	MLZ	18356	F	14.516667	-88.75	Honduras, Ocotepeque, Cerro Verde		X	
D. b. parva	MLZ	18361	M	14.516667	-88.75	Honduras, Ocotepeque, Cerro Verde		X	X
D. b. parva	MLZ	18362	M	14.516667	-88.75	Honduras, Ocotepeque, Cerro Verde		X	X
D. b. parva	MLZ	18363	M	14.516667	-88.75	Honduras, Ocotepeque, Cerro Verde		X	X
D. b. parva	MLZ	26635	M	14.33333	-87.4	Honduras, Francisco Morazán, Cantoral		X	X
D. b. parva	MLZ	26636	F	14.33333	-87.4	Honduras, Francisco Morazán, Cantoral		X	
D. b. parva	MLZ	26637	M	14.58333	-88	Honduras, Francisco Morazán, San Marcos de Guaimaca		X	

D. b. parva	LSUMZ	60978	ND	13.9388	-87.1691	Honduras, Francisco Morazán, Santa Ana	X		
D. b. parva	MVZ	86316	M	14.38306	-89.12899	Honduras, Ocotepeque, El Pital		X	X
D. b. parva	MVZ	86322	F	14.38306	-89.12899	Honduras, Ocotepeque, El Pital		X	
D. b. parva	AMNH	326509	M	14.220453	-87.913411	Honduras, La Paz, Muye		X	X
D. b. parva	AMNH	326510	F	14.236378	-87.957242	Honduras, Tegucigalpa, Archaga		X	
D. b. parva	AMNH	328423	M	14.33333	-87.4	Honduras, Francisco Morazán, Cantoral		X	
D. b. parva	AMNH	328424	M	14.33333	-87.4	Honduras, Francisco Morazán, Cantoral		X	X
D. b. parva	AMNH	328425	M	14.33333	-87.4	Honduras, Francisco Morazán, Cantoral		X	X
D. b. parva	AMNH	328426	M	14.33333	-87.4	Honduras, Francisco Morazán, Cantoral		X	X
D. b. parva	AMNH	328427	M	14.33333	-87.4	Honduras, Francisco Morazán, Cantoral		X	X
D. b. parva	AMNH	328428	M	14.3423	-87.2946	Honduras, Tegucigalpa, Archaga		X	X
D. b. parva	AMNH	328429	F	14.3423	-87.2946	Honduras, Tegucigalpa, Archaga		X	
D. b. parva	AMNH	328430	M	14.33333	-87.4	Honduras, Francisco Morazán, Cantoral		X	X
D. b. parva	USNM	348152	M	14.43333	-87.78333	Honduras, Francisco Morazán, Cantoral		X	X
D. b. parva	USNM	348172	F	14.43333	-87.78333	Honduras, Francisco Morazán, Cantoral		X	
D. plumbea	LSUMZ	28266	ND	ND	ND	Panama, Chiriquí	X		
E. hirundinacea	MZFC	MISA 110	M	19.7699444	-96.862083	Mexico, Veracruz, Villa Nueva	X		

Abbreviations for column names: Collection (Collec), Gender (G), Latitude (Lat), Longitude (Long), Genomic (Gen), Morphology (Morp), Coloration (Col).
Abbreviations for collections of voucher specimens: Colección Nacional de Aves, Instituto de Biología, UNAM (CNAV); Moore Laboratory of Zoology, Occidental College (MLZ); National Museum of Natural History, Smithsonian Institution (USNM), American Museum of Natural History (AMNH); Louisiana State University Museum of Natural Science (LSUMZ); Museum of Vertebrate Zoology, University of California, Berkeley (MVZ); University of Washington Burke Museum of Natural History and Culture (UWBM); Field Museum of Natural History (FMNH); Museo de Zoología “Alfonso L. Herrera”, Facultad de Ciencias, Universidad Nacional Autónoma de México (MZFC).
Abbreviations for Gender: female (F) and male (M).
The asterisk represents the reference genome used.
